# Prognostic Factors and Surgery for Breast Cancer Patients With Locoregional Recurrence: An Analysis of 5,202 Consecutive Patients

**DOI:** 10.3389/fonc.2021.763119

**Published:** 2021-10-13

**Authors:** Jiahui Huang, Yiwei Tong, Xiaosong Chen, Kunwei Shen

**Affiliations:** Department of General Surgery, Comprehensive Breast Health Center, Ruijin Hospital, Shanghai Jiaotong University School of Medicine, Shanghai, China

**Keywords:** breast cancer, risk factors, surgery, survival, locoregional recurrence

## Abstract

**Purpose:**

With the application of “less extensive surgery” in breast cancer treatment, the pattern of locoregional recurrence (LRR) has significantly changed. This study aims to evaluate the risk and prognostic factors of LRR in a recent large breast cancer cohort.

**Methods:**

Consecutive early breast cancer patients who received surgery from January 2009 to March 2018 in Shanghai Ruijin Hospital were retrospectively analyzed. LRR was defined as recurrence at the ipsilateral breast (IBTR), chest wall, or regional lymph nodes and without concurrent distant metastasis (DM). Patients’ characteristics and survival were compared among these groups.

**Results:**

Among 5,202 patients included, 87 (1.7%) and 265 (5.1%) experienced LRR and DM as first event after a median 47.0 (3.0–122.5) months’ follow-up. LRR was significantly associated with large tumor size and positive lymph node status (*p* < 0.05). Forty (46.0%) patients received further salvage surgery after LRR and had a significantly better 3-year post-recurrence overall survival than those who did not (94.7% *vs.* 60.7%, *p* = 0.012). Multivariate analysis showed that salvage surgery for LRR was independently associated with better survival (HR = 0.12, 95% CI 0.02–0.93, *p* = 0.043) along with estrogen receptor (ER) positivity (HR = 0.33, 95% CI 0.12–0.91, *p* = 0.033).

**Conclusion:**

LRR rate was relatively low in recent era of breast cancer treatment. Tumor size and lymph node status were associated with risk of LRR, and salvage surgery for selected LRR patients achieved an excellent outcome.

## Introduction

Breast cancer is the most commonly diagnosed cancer and the leading cause of cancer mortality in females worldwide (1). With a better understanding of tumor biologic behavior, innovations in screening techniques, and the development of comprehensive multidisciplinary treatment strategies, more breast cancers can be diagnosed at early stages. Less extensive surgery, for instance, breast-conserving surgery (BCS) followed by radiotherapy and sentinel lymph node biopsy (SLNB) in selected patients demonstrated equivalence with mastectomy and axillary lymph node dissection (ALND) in terms of survival but with less comorbidities (2, 3).

Locoregional recurrence (LRR) is a clinically relevant, predominant pattern of treatment failure in breast cancer. LRR patterns vary across initial surgical approach and mainly involve recurrence in chest wall post-mastectomy, residual breast after BCS, or regional lymph nodes (LNs). According to previous evidence, factors associated with increased risk of LRR include young age at diagnosis, greater tumor size, involvement of regional LN, high histological grade, triple negative (TN) or human epidermal growth factor receptor 2 (HER2)-positive subtype, lack of endocrine therapy, and omitting indicated adjuvant radiotherapy (4–6). Different from the palliative management of distant metastasis (DM), salvage surgery plays an important role in the comprehensive management of LRR. Patients who received salvage surgery for LRR reported relatively satisfactory 5-year overall survival (OS) ranging from 40.8% to 90.9% (7, 8), suggesting that selective LRR patients would benefit from salvage surgery and quite a number of LRR patients could be cured. However, retrospective series showed that between 15% and 37% patients with LRR had concurrent DM at the time of presentation (9–18). Disease outcomes and treatment strategies of these populations can be very different from those with LRR alone. The management of LRR should be based on systemic evaluation and be discussed in a multidisciplinary setting.

However, studies of LRR were mostly conducted in the late 1990s to early 2000s and in western populations. Following the change of initial surgical procedures from “maximal tolerable treatment” to “minimal effective treatment,” the pattern of LRR has also significantly changed. With an increasing rate of BCS and SLNB, now we meet more patients with ipsilateral breast tumor recurrence (IBTR) and regional LN recurrence in clinical practice. To this end, the objective of this study was to analyze the risk and prognostic factors of LRR in the current “less extensive surgery” era.

## Patients and Methods

### Patients

We retrospectively included consecutive female patients diagnosed with primary invasive breast cancer and received radical surgical treatment from January 2009 to March 2018 in Comprehensive Breast Health Center, Shanghai Ruijin Hospital. Patients with complete clinicopathological information, with at least 3 months of follow-up, were included in this study. Patient with *de novo* stage IV disease, with bilateral breast cancer, receiving neoadjuvant therapy for breast cancer, or with previous malignancy history were excluded from this study (**Supplementary Figure 1**). Patient baseline clinical characteristics were extracted from Shanghai Jiaotong University Breast Cancer Database (SJTU-BCDB).

### Pathological Assessment

Histopathological assessment and immunohistochemical (IHC) evaluation were conducted in the Department of Pathology, Ruijin Hospital, by at least two independent experienced pathologists. Estrogen receptor (ER) positivity and progesterone receptor (PR) positivity were defined as 1% or more positive invasive tumor cells with nuclear staining (19). HER2 status was first determined by IHC staining and scored as 0, 1+, 2+, and 3+ according to the American Society of Clinical Oncology/College of American Pathologists (ASCO/CAP) guideline (20). Samples with HER2 IHC 2+ were further examined by fluorescence *in situ* hybridization (FISH). HER2 positivity was defined as HER2 IHC 3+ or FISH positive. Five breast cancer molecular subtypes were classified according to the 2013 St. Gallen breast cancer consensus (21): Luminal A (ER+/HER2–, Ki67 < 14%, and PR ≥ 20%), Luminal B HER2− (ER+/HER2−, Ki67 ≥ 14%, or ER+/HER2−, PR < 20%, or ER−/PR+/HER2−), Luminal B HER2+ (ER or PR+/HER2+), TN (ER−/PR−/HER2–), and HER2 enriched (ER−/PR−/HER2+).

### Follow-Up and Disease Outcomes

Follow-up was accomplished annually by specialized breast cancer nurses in our center through outpatient medical history and/or phone calls. Recurrences in ipsilateral breast, chest wall, or regional LN (ipsilateral axillary, infra- and/or supraclavicular, or internal mammary LN) were considered LRR. DM included metastases to distant LN, bone, brain, liver, lung (including pleura and lymphangitic carcinomatosis), or others (including peritoneal, other organs not elsewhere classified, and skin not in the breast and chest wall). Patients with concurrent LRR and DM were categorized as DM as first recurrence event.

Recurrence-free interval (RFI) was defined as time from the date of breast cancer surgery to the date of first recurrence event. OS was defined as time from the date of breast cancer surgery to the date of death from any cause. Post-recurrence OS (PR-OS) was defined as the time from the date of first recurrence diagnosis to the time of death from any cause.

### Statistical Analysis

Patients were categorized into three groups according to their recurrence status, i.e., recurrence-free, LRR, and DM groups. Descriptive characteristics of categorical variables were tested using chi-squared test or Fisher’s exact test. Binary or multinomial logistic regression analysis was conducted to compare baseline clinicopathological features and adjuvant therapy among groups. Survival curves were plotted using the Kaplan–Meier method and compared between groups by log-rank test. Multivariate Cox proportional-hazards regression analyses were performed to calculate hazard ratios (HRs) and 95% confidence interval (CI) for recurrence and survival. All analyses were performed using IBM SPSS 22.0 (IBM Inc., Armonk, USA). All reported *p*-values were two-sided, and *p* < 0.05 was considered statistically significant.

## Results

### Patient Baseline Characteristics

A total of 5,202 women were included in this study. The median age was 55 (range: 22–93) years. Patients’ baseline clinicopathological characteristics at initial diagnosis and treatment for primary breast cancer were summarized in **Table 1**. Four thousand four hundred fifty-four (85.6%) patients had invasive ductal carcinoma, and 1,723 (33.1%) had node-positive disease. ER positivity were identified in 3,769 (72.5%) patients, and 1,181 (22.7%) had HER2-positive disease. With regard to local and systemic treatment, BCS was performed in 1,597 (30.7%) patients, while others received mastectomy as initial surgery for breast cancer. Two thousand five hundred sixty-three (49.3%) patients received SLNB, 2,598 (49.9%) patients received ALND, and the remaining 41 (0.8%) patients did not receive surgery for the axilla. Adjuvant radiotherapy was performed in 2,539 patients, including 86.4% of patients who underwent BCS and in 32.1% of patients who received mastectomy.

**Table 1:** Clinicopathological characteristics at initial diagnosis and treatment for primary breast cancer by different first recurrence events.

	Total	Recurrence-free	LRR	DM	*p* ^a^
	n	n (%)	n (%)	n (%)	
Age					0.066
<50 years	1,835	1,701 (92.7)	41 (2.2)	93 (5.1)	
≥50 years	3,367	3,149 (93.5)	46 (1.4)	172 (5.1)	
Menopausal status					0.526
Pre-menopausal	2,101	1,961 (93.3)	39 (1.9)	101 (4.8)	
Post-menopausal	3,101	2,889 (93.2)	48 (1.5)	164 (5.3)	
Tumor size					<0.001
≤2 cm	3,067	2,936 (95.7)	40 (1.3)	91 (3.0)	
>2 cm	2,020	1,804 (89.3)	47 (2.3)	169 (8.4)	
NA*	115	110 (95.7)	0 (0.0)	5 (4.3)	
Pathological type					0.015
IDC	4,454	4,135 (92.8)	76 (1.7)	243 (5.5)	
ILC	149	138 (92.6)	2 (1.3)	9 (6.0)	
Other invasive cancer	599	577 (96.3)	9 (1.5)	13 (2.2)	
Histological grade					<0.001
I–II	2,528	2,402 (95.0)	29 (1.1)	97 (3.8)	
III	1,896	1,711 (90.2)	43 (2.3)	142 (7.5)	
NA*	778	737 (94.7)	15 (1.9)	26 (3.3)	
Lymph node status					<0.001
Negative	3,440	3,307 (96.1)	41 (1.2)	92 (2.7)	
Positive	1,723	1,512 (87.8)	43 (2.5)	168 (9.8)	
NA*	39	31 (79.5)	3 (7.7)	5 (12.8)	
ER					<0.001
Positive	3,769	3,553 (94.2)	51 (1.4)	165 (4.4)	
Negative	1,424	1,288 (90.4)	36 (2.5)	100 (7.0)	
NA*	9	9 (100.0)	0 (0.0)	0 (0.0)	
PR					<0.001
Positive	3,099	2,951 (95.2)	34 (1.1)	114 (3.7)	
Negative	2,091	1,887 (90.3)	53 (2.5)	151 (7.2)	
NA*	9	9 (100.0)	0 (0.0)	0 (0.0)	
HER2					0.403
Negative	3,797	3,553 (93.5)	56 (1.5)	188 (5.0)	
Positive	1,181	1,097 (92.9)	24 (2.0)	60 (5.1)	
NA*	215	191 (88.9)	7 (3.3)	17 (7.9)	
Ki67					<0.001
≤20%	2,734	2,587 (94.6)	37 (1.4)	110 (4.0)	
>20%	2,428	2,224 (91.6)	50 (2.1)	154 (6.3)	
NA*	40	39 (97.5)	0 (0.0)	1 (2.5)	
Molecular subtype					<0.001
Luminal A	922	897 (97.3)	6 (0.7)	19 (2.0)	
Luminal B HER2−	2,082	1,940 (93.2)	35 (1.7)	107 (5.1)	
Luminal B HER2+	567	542 (95.6)	5 (0.9)	20 (3.5)	
HER2 enriched	614	555 (90.4)	19 (3.1)	40 (6.5)	
TN	725	652 (90.0)	15 (2.1)	58 (8.0)	
NA*	292	264 (90.4)	7 (2.4)	21 (7.2)	
Surgery of the breast					<0.001
BCS	1,597	1,513 (94.7)	36 (2.3)	48 (3.0)	
Mastectomy	3,605	3,337 (92.6)	51 (1.4)	217 (6.0)	
Surgery of the axilla					<0.001
SLNB	2,563	2,491 (97.2)	33 (1.3)	39 (1.5)	
ALND	2,598	2,326 (89.5)	51 (2.0)	221 (8.5)	
No surgery	41	33 (80.5)	3 (7.3)	5 (12.2)	
Adjuvant chemotherapy					<0.001
No	1,636	1,561 (95.4)	33 (2.0)	42 (2.6)	
Yes	3,550	3,279 (92.4)	54 (1.5)	217 (6.1)	
NA*	16	10 (62.5)	0 (0.0)	6 (37.5)	
Adjuvant radiotherapy					0.001
No	2,647	2,500 (94.4)	58 (2.2)	89 (3.4)	
Yes	2,539	2,340 (92.2)	57 (2.2)	142 (5.6)	
NA*	16	10 (62.5)	0 (0.0)	6 (37.5)	
Adjuvant targeted therapy					0.277
No	4,319	4,021 (93.1)	73 (1.7)	225 (5.2)	
Yes	867	819 (94.5)	14 (1.6)	34 (3.9)	
NA*	16	10 (62.5)	0 (0.0)	6 (37.5)	
Adjuvant endocrine therapy					<0.001
No	1,570	1,418 (90.3)	44 (2.8)	108 (6.9)	
Yes	3,616	3,422 (94.6)	43 (1.2)	151 (4.2)	
NA*	16	10 (62.5)	0 (0.0)	6 (37.5)	

LRR, locoregional recurrence; DM, distant metastasis; IDC, invasive ductal carcinoma; ILC, invasive lobular carcinoma; NA, not available; ER, estrogen receptor; PR, progesterone receptor; HER2, human epidermal growth factor receptor 2; TN, triple negative; BCS, breast-conserving surgery; SLNB, sentinel lymph node biopsy; ALND, axillary lymph node dissection.

a Compared between groups by chi-square test.

^*^Variable NA was not included in the analysis.

### Patient Characteristics Associated With First Recurrence Event

After a median follow-up of 47.0 (range: 3.0–122.5) months, 352 (6.8%) patients experienced breast cancer recurrence, including 87 (1.7%) LRR and 265 (5.1%) DM as first recurrence event. The 5-year estimated LRR rate was 2.2% in the whole population: 3.3% in patients receiving BCS and 1.7% in patients receiving mastectomy. Tumor size, pathological type, histological grade, LN status, ER status, PR status, Ki67 level, molecular subtype, surgery of the breast, surgery of the axilla, adjuvant chemotherapy, adjuvant radiotherapy, and adjuvant endocrine therapy were differently distributed among patients with no recurrence, LRR, and DM in the univariate model (all *p* < 0.05; **Table 1**), while no difference was observed in age, menopausal status, HER2 status, or adjuvant targeted therapy among three groups (*p* > 0.05).

Multivariate analysis demonstrated that tumor size (*p* < 0.001; **Table 2**), histological grade (*p* < 0.001), lymph node status (*p* < 0.001), molecular subtype (*p* = 0.005), surgery of the breast (*p* < 0.001), surgery of the axilla (*p* < 0.001), and adjuvant chemotherapy (*p* = 0.013) were independently associated with first recurrence events. Comparison between patients with LRR and recurrence-free showed that tumor size >2.0 cm (OR = 2.13, 95% CI 1.31–3.48, *p* = 0.002), positive LNs (OR = 3.24, 95% CI 1.75–6.02, *p* < 0.001), primary BCS (OR = 3.04, 95% CI 1.73–5.33, *p* < 0.001), not receiving adjuvant chemotherapy (OR = 2.48, 95% CI 1.37–4.50, *p* = 0.003), and not receiving adjuvant radiotherapy (OR = 1.91, 95% CI 1.07–3.42, *p* = 0.030) were independent risk factors for LRR. Regarding patients with DM as first recurrence event, LRR patients had higher rates of BCS (OR = 3.86, 95% CI 1.96–7.58, *p* < 0.001), SLNB (OR = 2.80, 95% CI 1.37–5.75, *p* = 0.005), not receiving adjuvant chemotherapy (OR = 2.81, 95% CI 1.37–5.75, *p* = 0.013), and not receiving adjuvant radiotherapy (OR = 2.52, 95% CI 1.21–5.20, *p* = 0.042).

**Table 2:** Multivariate logistic regression of predictors for disease recurrence type*.

	Recurrence-free		Distant metastasis		*p*
	OR (95% CI)	*p*	OR (95% CI)	*p*	
Tumor size					<0.001
>2 cm	1.0		1.0		
≤2 cm	2.13 (1.31–3.48)	0.002	1.12 (0.64–1.97)	0.686	
Pathological type					0.182
IDC	1.0		1.0		
ILC	1.61 (0.28–9.40)	0.593	2.40 (0.32–18.02)	0.394	
Other invasive cancer	2.12 (0.63–7.05)	0.222	1.15 (0.27–4.93)	0.846	
Histological grade					<0.001
I-II	1.0		1.0		
III	0.66 (0.38–1.15)	0.145	0.98 (0.53–1.84)	0.961	
NA	0.41 (0.13–1.25)	0.116	0.53 (0.14–2.07)	0.361	
Lymph node status					0.048
Negative	3.24 (1.75–6.02)	<0.001	1.82 (0.89–3.73)	0.103	
Positive	1.0		1.0		
Molecular subtype					0.005
Luminal A	1.0		1.0		
Luminal B HER2−	0.41 (0.16–1.01)	0.052	0.78 (0.27–2.25)	0.642	
Luminal B HER2+	0.89 (0.23–3.40)	0.862	0.96 (0.21–4.32)	0.959	
HER2 enriched	0.34 (0.10–1.20)	0.094	0.55 (0.12–2.48)	0.440	
TN	0.52 (0.14–1.88)	0.316	1.30 (0.29–5.87)	0.730	
Surgery of the breast					<0.001
BCS	1.0		1.0		
Mastectomy	3.04 (1.73–5.33)	<0.001	3.86 (1.96–7.58)	<0.001	
Surgery of the axilla					<0.001
SLNB	1.0		1.0		
ALND	0.84 (0.46–1.51)	0.552	2.80 (1.37–5.75)	0.005	
Adjuvant chemotherapy					0.013
No	1.0		1.0		
Yes	2.48 (1.37–4.50)	0.003	2.81 (1.37–5.75)	0.013	
Adjuvant radiotherapy					0.090
No	1.0		1.0		
Yes	1.91 (1.07–3.42)	0.030	2.52 (1.21–5.20)	0.042	
Adjuvant endocrine therapy					
No	1.0		1.0		
Yes	2.14 (0.94–4.83)	0.069	1.64 (0.62–4.35)	0.320	

OR, odds ratio; CI, confidence interval; LRR, locoregional recurrence; DM, distant metastasis; IDC, invasive ductal carcinoma; ILC, invasive lobular carcinoma; NA, not available; HER2, human epidermal growth factor receptor 2; TN, triple negative; BCS, breast-conserving surgery; SLNB, sentinel lymph node biopsy; ALND, axillary lymph node dissection.

^*^Reference category was LRR group.

### Factors Influencing Salvage Surgery for Locoregional Recurrence Patients

Forty out of 87 (46.0%) LRR patients received further salvage surgery. **Table 3** summarizes the clinicopathological features associated with the reception of salvage surgery in LRR patients. Age at recurrence, primary tumor size, primary lymph node status, primary surgery of the breast and axilla, and LRR type significantly influenced the choice of surgery for LRR (*p* < 0.05; **Table 3**). Patients with IBTR received more salvage surgery as compared with LRR patients with chest wall recurrence or reginal LN recurrence (*p* < 0.001). Twenty-one out of 26 (80.8%) patients with IBTR received salvage surgery, all of whom received mastectomy with or without ALND. Only five patients with isolated IBTR did not receive surgery for LRR, including two patients refusing further treatment, two treated with endocrine therapy but not surgery due to advanced age, and one participating in a clinical trial of a new drug. Twelve out of 27 (44.4%) patients with chest wall recurrence received extended tumor excision, while seven out of 34 (20.6%) patients with regional LN recurrence received LN dissection surgery. Among 27 patients who did not receive surgery for regional LN recurrence, nine, 17, and one patients were with ALN recurrence, supraclavicular/infraclavicular LN recurrence, and internal mammary LN recurrence.

**Table 3:** Univariate analysis for clinicopathological features related to salvage surgery decision for LRR patients.

	No surgery	Surgery	p^a^
	n (%)	n (%)	
Age at primary diagnosis			0.074
<50 years	18 (43.9)	23 (56.1)	
≥50 years	29 (63.0)	17 (37.0)	
Age at recurrence			0.028
<70 years	37 (49.3)	38 (50.7)	
≥70 years	10 (83.3)	2 (16.7)	
Menopausal status at primary diagnosis			0.078
Pre-menopausal	17 (43.6)	22 (56.4)	
Post-menopausal	30 (62.5)	18 (37.5)	
Tumor size^*^			0.004
≤2 cm	15 (37.5)	25 (62.5)	
>2 cm	32 (68.1)	15 (31.9)	
Pathological type^*^			0.970
IDC	41 (53.9)	35 (46.1)	
Other invasive cancer	6 (54.5)	5 (45.5)	
Histological grade^*^			0.078
I–II	14 (48.3)	15 (51.7)	
III	28 (65.1)	15 (34.9)	
NA	5 (33.3)	10 (66.7)	
Lymph node status^*^			<0.001
Negative	14 (34.1)	27 (65.9)	
Positive	33 (76.7)	10 (23.3)	
NA^†^	0 (0.0)	3 (100.0)	
ER^*^			0.498
Positive	21 (58.3)	15 (41.7)	
Negative	26 (51.0)	25 (49.0)	
PR^*^			0.871
Positive	29 (54.7)	24 (45.3)	
Negative	18 (52.9)	16 (47.1)	
HER2^*^			0.461
Negative	33 (58.9)	23 (41.1)	
Positive	12 (50.0)	12 (50.0)	
NA^†^	2 (28.6)	5 (71.4)	
Ki67^*^			0.387
≤20%	18 (48.6)	19 (51.4)	
>20%	29 (58.0)	21 (42.0)	
Molecular subtype^*^			0.447
Luminal A	2 (33.3)	4 (66.7)	
Luminal B HER2−	20 (57.1)	15 (42.9)	
Luminal B HER2+	2 (40.0)	3 (60.0)	
HER2 enriched	10 (52.6)	9 (47.4)	
TN	11 (73.3)	4 (26.7)	
NA^†^	2 (28.6)	5 (71.4)	
Primary surgery of the breast			0.001
BCS	12 (33.3)	24 (66.7)	
Mastectomy	35 (68,6)	16 (31.4)	
Primary surgery of the axilla			0.001
SLNB	11 (33.3)	22 (66.7)	
ALND	36 (70.6)	15 (29.4)	
No surgery	0 (0.0)	3 (100.0)	
LRR type			<0.001
IBTR	5 (19.2)	21 (80.8)	
Chest wall	15 (55.6)	12 (44.4)	
LNR	27 (79.4)	7 (20.6)	
RFI			0.246
≤24 months	21 (61.8)	13 (38.2)	
>24 months	26 (49.1)	27 (50.9)	

LRR, locoregional recurrence; IDC, invasive ductal carcinoma; ILC, invasive lobular carcinoma; NA, not available; ER, estrogen receptor; PR, progesterone receptor; HER2, human epidermal growth factor receptor 2; TN, triple negative; BCS, breast-conserving surgery; SLNB, sentinel lymph node biopsy; ALND, axillary lymph node dissection; IBTR, ipsilateral breast tumor recurrence; LNR, lymph node recurrence; DM, distant metastasis; RFI, recurrence-free interval.

a Compared between groups by chi-square test.

^*^Tumor characteristics were from primary breast cancer.

^†^Variable NA was not included in the analysis.

Multivariate analysis showed that primary tumor size (*p* = 0.039), primary surgery of the axilla (*p* = 0.006), and LRR type (*p* < 0.001) were factors that independently influenced the choice of surgery for LRR (**Table 4**). Patients with smaller primary tumor size, primary SLNB, and IBTR had significantly higher probability to receive surgical treatment for LRR. Patients with regional LN recurrence were less likely to receive surgery for LRR than were patients with IBTR only (OR = 0.07, 95% CI 0.02–0.30, *p* < 0.001), while the probability of surgery for LRR was comparable between patients with chest wall recurrence and IBTR (OR = 0.36, 95% CI 0.09–1.47, *p* = 0.155).

**Table 4:** Multivariate analysis for clinicopathological features related to salvage surgery decision for LRR patients.

	Multivariate analysis	
	OR (95% CI)	*p*
Age at recurrence (<70 *vs.* ≥ 70 years)	5.37 (0.58–50.14)	0.140
Tumor size^*^ (<2 *vs.* ≥2 cm)	3.29 (1.06–10.17)	0.039
Lymph node status^*^ (negative *vs.* positive)	2.03 (0.52–8.00)	0.312
Primary surgery of the breast (BCS *vs.* mastectomy)	0.48 (0.06–3.75)	0.484
Primary surgery of the axilla (SLNB *vs.* ALND)	5.01 (1.60–15.68)	0.006
LRR type		
Chest wall only *vs.* IBTR only	0.36 (0.09–1.47)	0.155
Regional LNR only *vs.* IBTR only	0.07 (0.02–0.30)	<0.001

LRR, locoregional recurrence; OR, odds ratio; CI, confidence interval; BCS, breast-conserving surgery; SLNB, sentinel lymph node biopsy; ALND, axillary lymph node dissection; IBTR, ipsilateral breast tumor recurrence; LNR, lymph node recurrence.

^*^Tumor characteristics were from primary breast cancer. Variable NA was not included in multivariate analysis.

### Survival Outcome With Different Recurrence Events

**Figure 1:**
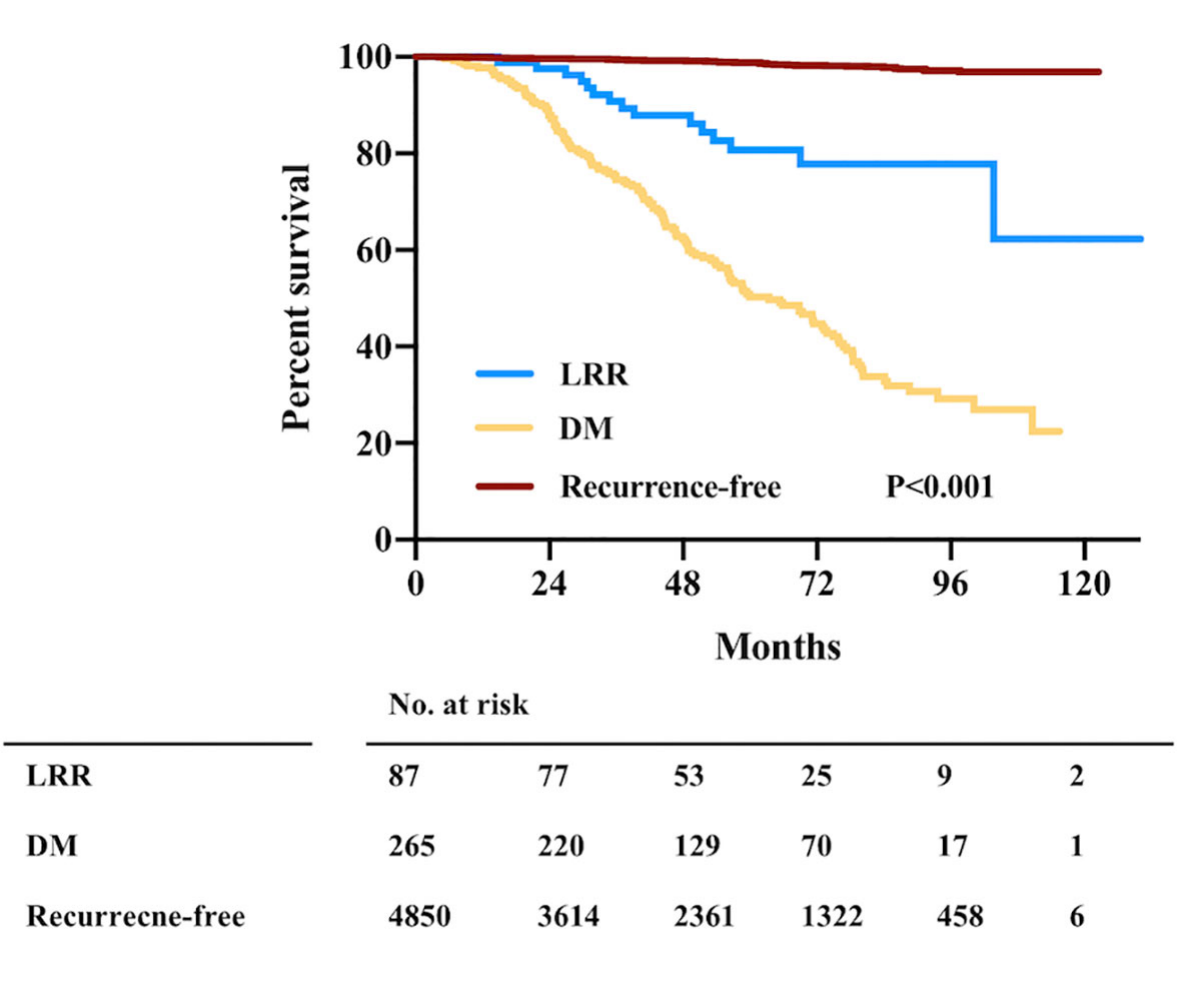
Overall survival by first recurrence event in the whole population. LRR, locoregional recurrence; DM, distant metastasis; No., number.

**Figure 2:**
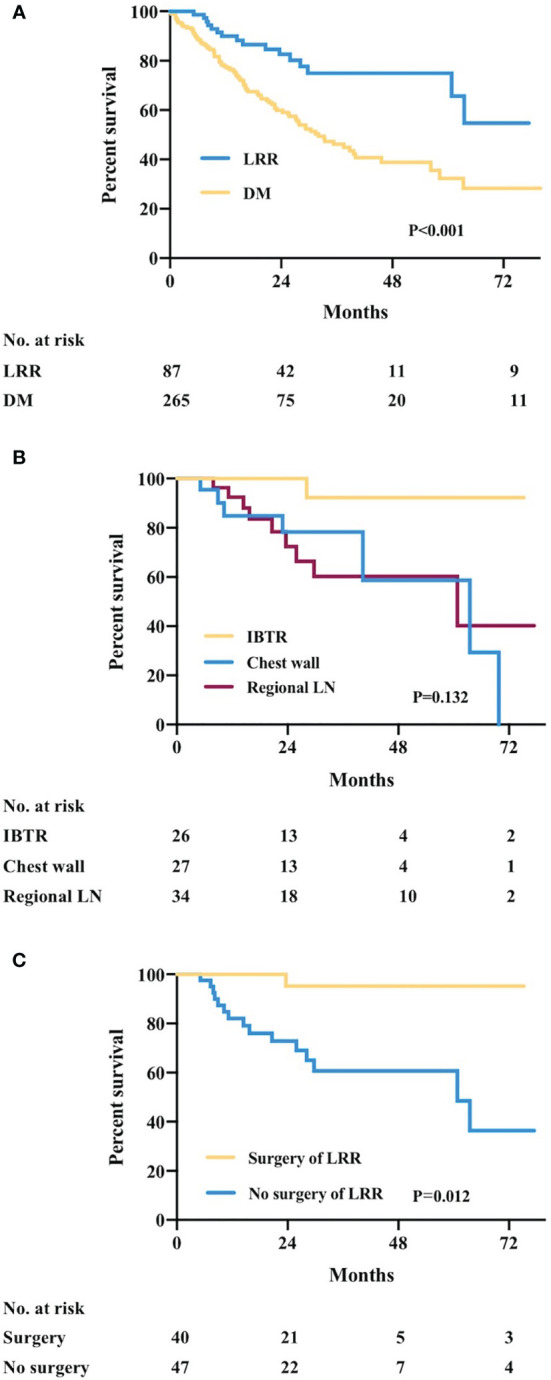
Post-recurrence overall survival (PR-OS) by recurrence type. **(A)** PR-OS in recurrent patients by first recurrence event. **(B)** PR-OS in LRR patients by LRR type. **(C)** PR-OS in LRR patients receiving or not salvage surgery for LRR. LRR, locoregional recurrence; DM, distant metastasis; No., number; IBTR, ipsilateral breast tumor recurrence; LN, lymph node.

The estimated 5-year OS was 80.7%, 50.3%, and 98.8% for patients with LRR, patients with DM, and recurrence-free patients, respectively (*p* < 0.001, **Figure 1**). Among the 87 patients with LRR, 26, 27, and 34 patients had IBTR, chest wall recurrence, and LN recurrence, respectively. During a median post-recurrence follow-up time of 21.3 (range: 1.0–77.5) months, 30 deaths were recorded. PR-OS curve is shown in **Figure 2A**. Patients with LRR as first event had a significantly better PR-OS than those with DM (3-year PR-OS 75.0% *vs.* 37.1%; *p* < 0.001, **Figure 2A**).

Univariate analysis showed that primary tumor size (*p* = 0.033; **Supplementary Table 1**), primary ER status (*p* = 0.033), primary surgery of the axilla (*p* = 0.034), LRR type (regional LN *vs.* IBTR only, *p* = 0.045), and surgery of LRR (*p* = 0.012) were factors associated with PR-OS. The estimated 3-year PR-OS was 90.9%, 77.3%, and 60.3% in patients with recurrence type of IBTR, chest wall, and regional LN, respectively (*p* = 0.132, **Figure 2B**). The estimated 3-year PR-OS was 94.7% in patients receiving surgery after LRR, which was significantly higher than that not receiving surgery (60.7%, *p* = 0.012, **Figure 2C**). In multivariate analysis, ER positivity (HR = 0.33, 95% CI 0.12–0.91, *p* = 0.033) and salvage surgery of LRR (HR = 0.11, 95% CI 0.02–0.93, *p* = 0.043) were independently associated with better PR-OS for LRR patients (**Table 5**).

**Table 5:** Multivariate analysis of factors associated with post-recurrence overall survival in patients with locoregional recurrence.

	Multivariate analysis	
	HR (95% CI)	*p*
Tumor size^*^ (>2 *vs*. ≤2 cm)	1.06 (0.25–4.63)	0.934
ER^*^ (positive *vs*. negative)	0.33 (0.12–0.91)	0.033
Primary surgery of the axilla (ALND *vs*. SLNB)	6.44 (0.83–49.63)	0.074
LRR type		0.496
Chest wall *vs*. IBTR only	3.52 (0.38–32.72)	0.262
LNR *vs*. IBTR only	2.40 (0.26–21.85)	0.337
Surgery of LRR (Yes *vs*. No)	0.12 (0.02–0.93)	0.043

HR, hazard ratio; CI, confidence interval; ER, estrogen receptor; ALND, axillary lymph node dissection; SLNB, sentinel lymph node biopsy; LRR, locoregional recurrence; IBTR, ipsilateral breast tumor recurrence; LNR, lymph node recurrence.

^*^Tumor characteristics were from primary breast cancer. Patients with unknown lymph node status were not included in multivariate analysis.

## Discussion

In this cohort of 5,202 consecutive breast cancer patients, we showed that LRR after radical surgery in the modern era is relatively low. Clinicopathological factors, including large tumor size, positive lymph node status, and molecular subtype, were significantly associated with increased risk of LRR. Primary surgical treatment for breast or adjuvant chemotherapy or radiotherapy also influenced the risk of LRR. Moreover, LRR patients had higher rates of receiving BCS or SLNB and not receiving adjuvant chemotherapy or radiotherapy compared with DM patients. Furthermore, we found that LRR types were related with salvage surgery choice after LRR. For patients receiving surgery after LRR, they could achieve an excellent outcome after recurrence.

According to the Early Breast Cancer Trialists’ Collaborative Group (EBCTCG) overview, which included trials up to year 2000 evaluating the effects of radiotherapy, the 5-year LRR rate was 7% in patients after BCS and radiotherapy and 6% in patients after mastectomy (22). A reduction of LRR has been seen in the recent years with the improvement in imaging, earlier diagnosis, surgical planning, and adjuvant therapy for breast cancer patients (5). In our study, the 5-year LRR rate was 2.8% in the whole population: 3.8% in patients receiving BCS and 2.5% in patients receiving mastectomy, which were quite low compared with the established evidence. The low LRR rate highlights the effect of multiple changes in breast cancer management over the past two decades.

Several clinicopathological factors as well as treatment patterns were associated with LRR after surgery in early breast cancer patients. Not surprisingly, in our study, we found that large tumor size, positive LN status, and primary BCS were identified as independent risk factors for LRR, which was consistent with previous studies (23, 24). Meanwhile, adjuvant chemotherapy and radiotherapy can effectively reduce the risk of LRR. Neoadjuvant chemotherapy was one of risk factors for local recurrence as reported by the EBCTCG meta-analysis (25), but neoadjuvant population was not included in our study. There was controversy in grouping patients when analyzing the two populations together, since there is discordance of molecular biomarkers before and after neoadjuvant therapy, and the staging of patients will change after neoadjuvant therapy. Also, in neoadjuvant study, we usually use event-free survival to evaluate patients’ outcome, which includes more information than recurrence-free interval that we evaluated in adjuvant studies. By reason of the foregoing, we excluded patients who received neoadjuvant therapy in this study, to make the evaluation standardized in the whole study population.

We also found that LRR was a less common recurrence event, as either first recurrence event or subsequent recurrence event comparing with DM. Few studies directly compared the difference between patients with different first recurrence events. Our study demonstrated that LRR patients had higher rate of receiving primary BCS, primary SLNB, and lower rate of receiving adjuvant chemotherapy or radiotherapy, indicating that more effective systemic and local treatment should be evaluated to further reduce the rate of LRR.

In the modern era of breast cancer treatment, management of LRR breast cancer patients remained a big challenge due to lower LRR events, fewer high quality clinical evidence, and relatively hard to follow-up patients. For patients who developed IBTR after BCS, the current standard of care is further salvage surgery, including salvage mastectomy or repeat BCS (26), which can achieve 59% to 90.9% 5-year OS after salvage surgery (11, 27–30). There is also another special consideration for patients with IBTR that whether it is “true recurrence” or “new primary,” since new primaries should theoretically have a prognosis independent of the primary breast cancer. The rate of new primary breast cancer in patients with IBTR was 18%–58.9% in published studies (31–34), also strengthening the reason for surgery of IBTR. For patients with isolated chest wall recurrence, full-thickness chest wall resection can be performed with excellent survival and low morbidity. In a recent systematic meta-analysis of 48 studies accounting for 1,305 patients who received full-thickness resection for chest wall recurrence, the mortality was consistently low (<1%), and 5-year OS was 40.8% (8). Axillary recurrence rates are rare, ranging of 1% to 3% after adequate management of primary disease (35, 36). Salvage ALND was the first choice for selected patients and can be performed in 45.5% to 69.5% patients (37, 38). Surgery of LRR might be encouraged in patients who can achieve R0 resection. In our study, salvage surgery was performed in 46.0% of LRR patients: 80.8% for IBTR, 44.4% for chest wall recurrence, and 20.6% for regional LN recurrence. Patients with smaller primary tumor, receiving primary SLNB, and LRR type were related with the choice of surgery after LRR. Although the post-LRR follow-up period is short, and there was selective bias in patients receiving salvage surgery, we do observe that patients receiving surgery for LRR achieved a better PR-OS, which emphasized the importance of surgery as part of multidisciplinary management of LRR patients.

Some limitations of this study exist. The data were collected retrospectively, which may have led to selection bias. The follow-up time is relatively short, and only a small number of LRR events were recorded, given that LRR was less common in clinical practice. The actual site of recurrence may influence the possibility of surgery for LRR lesions and were not analyzed in this study. Details of the recurrence including site and pathologic features of the recurrent lesion are not completely collected, and we cannot distinguish whether there is true recurrence or new primary breast cancer in patients with IBTR. Treatments of LRR out of surgery such as systemic therapy or radiotherapy and their impact on survival were not recorded or analyzed in this study. More comprehensive treatment data as well as longer follow-up are warranted to find the best management for LRR patients.

## Conclusion

LRR rate was relatively low in the modern era of breast cancer treatment cohort. Large tumor size, positive lymph node status, and treatment strategies were associated with LRR. Moreover, LRR patients had a higher rate of receiving primary BCS or SLNB, and not receiving adjuvant chemotherapy or radiotherapy compared with DM patients. LRR patients treated with salvage surgery experienced excellent survival, indicating salvage surgery should play an important role in multidisciplinary treatment of LRR patients.

## Data Availability Statement

The raw data supporting the conclusions of this article will be made available by the authors, without undue reservation.

## Ethics Statement

The studies involving human participants were reviewed and approved by Ethical Committees of Ruijin Hospital, Shanghai Jiaotong University School of Medicine. Written informed consent for participation was not required for this study in accordance with the national legislation and the institutional requirements.

## Author Contributions

All authors listed have made a substantial, direct, and intellectual contribution to the work and approved it for publication.

## Funding

This study was funded by the National Natural Science Foundation of China (81772797, 82072937); Shanghai Municipal Education Commission—Gaofeng Clinical Medicine Grant Support (20172007); Shanghai Jiao Tong University Yi Gong Jiao Cha Funding (YG2019QNA30); and Ruijin Hospital, Shanghai Jiao Tong University School of Medicine—”Guangci Excellent Youth Training Program” (GCQN-2019-B07). All these financial sponsors had no role in the study design, data collection, analysis, or interpretation.

## Conflict of Interest

The authors declare that the research was conducted in the absence of any commercial or financial relationships that could be construed as a potential conflict of interest.

## Publisher’s Note

All claims expressed in this article are solely those of the authors and do not necessarily represent those of their affiliated organizations, or those of the publisher, the editors and the reviewers. Any product that may be evaluated in this article, or claim that may be made by its manufacturer, is not guaranteed or endorsed by the publisher.
