# Supplementary material for: Prognostic Factors and Surgery for Breast Cancer Patients With Locoregional Recurrence: An Analysis of 5,202 Consecutive Patients
Source: Front Oncol. 2021 Oct 13;11:763119. doi: 10.3389/fonc.2021.763119 (PMC8548583; doi:10.3389/fonc.2021.763119)
Supplement: Supplementary file 1 [file DataSheet_1.docx]

Supplementary Material

# Supplementary Figures and Tables

## Supplementary Figures


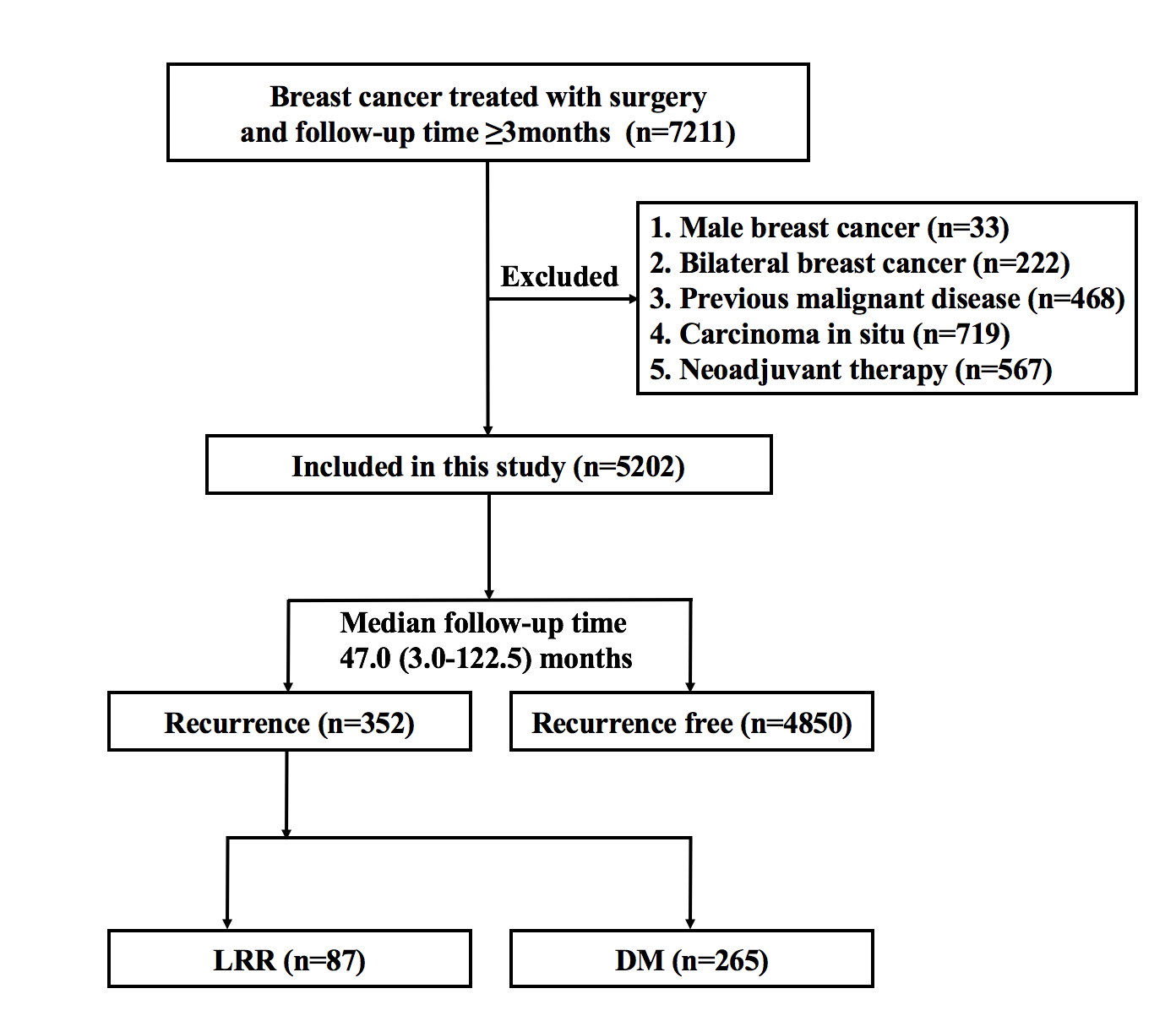


**Supplementary Figure 1.** Study flowchart.

Abbreviations: LRR, locoregional recurrence; DM, distant metastasis.

## Supplementary Tables

**Supplementary Table 1. Univariate analysis of factors associated with post-recurrence overall survival in patients with locoregional recurrence**

|  |  | HR(95%CI) |  | *P* |  |
| --- | --- | --- | --- | --- | --- |
| Age (≥ 50 years vs < 50 years) |  | 1.18(0.44-3.18) |  | 0.746 |  |
| Menopausal status (Post vs Pre) |  | 1.48(0.54-4.08) |  | 0.450 |  |
| Tumor size^*^ (> 2cm vs ≤ 2cm) |  | 3.46(1.10-10.87) |  | 0.033 |  |
| Pathological type^*^ |  |  |  | 0.208 |  |
| ILC vs IDC |  | 5.04(0.63-40.54) |  | 0.129 |  |
| Other invasive cancer vs IDC |  | 2.01 (0.57-7.13) |  | 0.281 |  |
| Histological Grade^*^ |  |  |  | 0.107 |  |
| III vs I-II |  | 4.55(1.00-20.72) |  | 0.050 |  |
| NA vs I-II |  | 2.09(0.35-12.57) |  | 0.422 |  |
| Lymph node status^*^ (Positive vs Negative) |  | 3.09(0.99-9.60) |  | 0.051 |  |
| ER^*^ (Negative vs Positive) |  | 3.03(1.09-8.40) |  | 0.033 |  |
| PR^*^ (Negative vs Positive) |  | 1.54(0.50-4.78) |  | 0.456 |  |
| HER2 ^*^ (Positive vs Negative) |  | 0.35(0.08-1.55) |  | 0.167 |  |
| Ki67 ^*^ (>20% vs ≤20%) |  | 1.72(0.59-4.98) |  | 0.318 |  |
| Molecular subtype^*^ |  |  |  | 0.126 |  |
| Luminal B HER2- vs Luminal A |  | 5300.76(0.00-5.68E+81) |  | 0.925 |  |
| Luminal B HER2+ vs Luminal A |  | 0.98(0.00-5.20E+94) |  | 1.000 |  |
| HER2 enriched vs Luminal A |  | 5356.08(0.00-6.76E+81) |  | 0.925 |  |
| TN vs Luminal A |  | 21502.72(0.00-2.30E+82) |  | 0.913 |  |
| Surgery of the breast (Mastectomy vs BCS) |  | 3.21(0.91-11.26) |  | 0.069 |  |
| Surgery of the axilla (ALND vs SLNB) |  | 8.94(1.18-67.83) |  | 0.034 |  |
| Adjuvant chemotherapy (Yes vs No) |  | 2.02(0.65-6.30) |  | 0.225 |  |
| Adjuvant radiotherapy (Yes vs No) |  | 1.30(0.48-3.50) |  | 0.602 |  |
| Adjuvant targeted therapy (Yes vs No) |  | 0.04(0.00-14.12) |  | 0.272 |  |
| Adjuvant endocrine therapy (Yes vs No) |  | 0.56(0.20-1.54) |  | 0.258 |  |
| RFI (≤24 months vs >24 months) |  | 1.79(0.67-4.77) |  | 0.247 |  |
| LRR type |  |  |  | 0.132 |  |
| Chest wall only vs IBTR only |  | 7.48(0.90-62.30) |  | 0.063 |  |
| Regional LN recurrence vs IBTR only |  | 8.26(1.04-65.35) |  | 0.045 |  |
| Surgery of LRR (Yes vs No) |  | 0.08(0.01-0.57) |  | 0.012 |  |

Abbreviations: ILC, invasive lobular carcinoma; IDC, invasive ductal carcinoma; NA, not available; ER, estrogen receptor; PR, progesterone receptor; HER2, human epidermal growth factor receptor 2; TN, triple negative; BCS, breast conserving surgery; ALND, axillary lymph node dissection; SLNB, sentinel lymph node biopsy; RFI, recurrence free interval; DM, distant metastasis; LRR, locoregional recurrence; IBTR, ipsilateral breast tumor recurrence; LNR, lymph node recurrence.

^*^Tumor characteristics were from primary breast cancer.

**Supplementary Table 2. Clinico-pathological characteristics of LRR patients with or without DM**

|  |  | LRR without DM | |  | LRR with DM | |  | Univariate analysis | | |  |
| --- | --- | --- | --- | --- | --- | --- | --- | --- | --- | --- | --- |
|  |  | n | % |  | n | % |  | OR(95%CI) |  | *P* |  |
| Age |  |  |  |  |  |  |  |  |  | 0.469 |  |
| < 50 years |  | 41 | 78.8 |  | 11 | 21.2 |  | 1 |  |  |  |
| ≥ 50 years |  | 46 | 73.0 |  | 17 | 27.0 |  | 1.38(0.58-3.28) |  |  |  |
| Menopausal status |  |  |  |  |  |  |  |  |  | 0.609 |  |
| Pre-menopausal |  | 39 | 70.9 |  | 16 | 29.1 |  | 1 |  |  |  |
| Post-menopausal |  | 48 | 80.0 |  | 12 | 20.0 |  | 0.61(0.26-1.44) |  |  |  |
| Tumor size |  |  |  |  |  |  |  |  |  | 0.342 |  |
| ≤ 2cm |  | 40 | 80.0 |  | 10 | 20.0 |  | 1 |  |  |  |
| > 2cm |  | 47 | 72.3 |  | 18 | 27.7 |  | 1.53(0.64-3.70) |  |  |  |
| Pathological type |  |  |  |  |  |  |  |  |  | 0.963 |  |
| IDC |  | 76 | 73.8 |  | 27 | 27 |  | 1 |  |  |  |
| ILC |  | 2 | 2 |  | 1 | 1 |  | 1.41(0.123-16.15) |  | 0.784 |  |
| Other invasive cancer |  | 9 | 9 |  | 0 | 0 |  | 0.00(0.00-NA) |  | 0.999 |  |
| Histological Grade |  |  |  |  |  |  |  |  |  | 0.263 |  |
| I-II |  | 29 | 72.5 |  | 11 | 27.5 |  | 1 |  |  |  |
| III |  | 43 | 72.9 |  | 17 | 27.1 |  | 0.98(0.40-2.41) |  | 0.967 |  |
| NA |  | 15 | 93.8 |  | 1 | 6.3 |  | 0.176(0.021-1.494) |  | 0.111 |  |
| Lymph node status |  |  |  |  |  |  |  |  |  | 0.862 |  |
| Negative |  | 41 | 77.4 |  | 12 | 22.6 |  | 1 |  |  |  |
| Positive |  | 43 | 72.9 |  | 16 | 27.1 |  | 1.271(0.537-3.011) |  | 0.585 |  |
| NA |  | 3 | 100.0 |  | 0 | 0.0 |  | 0.000(0.000-NA) |  | 0.999 |  |
| ER |  |  |  |  |  |  |  |  |  | 0.639 |  |
| Negative |  | 36 | 73.5 |  | 13 | 26.5 |  | 1 |  |  |  |
| Positive |  | 51 | 77.3 |  | 15 | 22.7 |  | 0.814(0.346-1.918) |  |  |  |
| PR |  |  |  |  |  |  |  |  |  | 0.985 |  |
| Negative |  | 53 | 75.7 |  | 17 | 24.3 |  | 1 |  |  |  |
| Positive |  | 34 | 75.6 |  | 11 | 24.4 |  | 1.009(0.422-2.413) |  |  |  |
| HER2 |  |  |  |  |  |  |  |  |  | 0.733 |  |
| Negative |  | 56 | 74.7 |  | 19 | 25.3 |  | 1 |  |  |  |
| Positive |  | 24 | 75.0 |  | 8 | 25.0 |  | 0.982(0.378-2.552) |  | 0.971 |  |
| NA |  | 7 | 87.5 |  | 1 | 12.5 |  | 0.421(0.049-3.647) |  | 0.432 |  |
| Ki67 |  |  |  |  |  |  |  |  |  | 0.192 |  |
| ≤20% |  | 37 | 82.2 |  | 8 | 17.8 |  | 1 |  |  |  |
| >20% |  | 50 | 71.4 |  | 20 | 28.6 |  | 1.850(0.73504.659) |  |  |  |
| Molecular subtype |  |  |  |  |  |  |  |  |  | 0.947 |  |
| Luminal A |  | 6 | 100.0 |  | 0 | 0.0 |  | 0.00(0.00-NA) |  | 0.999 |  |
| Luminal B HER2- |  | 35 | 74.5 |  | 12 | 25.5 |  | 0.74(0.24-2.23) |  | 0.587 |  |
| Luminal B HER2+ |  | 5 | 71.4 |  | 2 | 28.6 |  | 0.86(0.13-5.56) |  | 0.872 |  |
| HER2 enriched |  | 19 | 76.0 |  | 6 | 24.0 |  | 0.68(0.19-2.44) |  | 0.551 |  |
| TN |  | 15 | 68.2 |  | 7 | 31.8 |  | 1 |  |  |  |
| NA |  | 7 | 87.5 |  | 1 | 12.5 |  | 0.31(0.03-2.99) |  | 0.309 |  |
| RFI |  |  |  |  |  |  |  |  |  | 0.310 |  |
| ≤24 months |  | 34 | 70.8 |  | 14 | 29.2 |  | 1 |  |  |  |
| >24 months |  | 53 | 79.1 |  | 14 | 20.9 |  | 0.64(0.27-1.51) |  |  |  |
| LRR type |  |  |  |  |  |  |  |  |  | 0.015 |  |
| IBTR only |  | 26 | 89.7 |  | 3 | 10.3 |  | 0.20(0.05-0.73) |  | 0.015 |  |
| Chest wall only |  | 27 | 84.4 |  | 5 | 15.6 |  | 0.32(0.11-0.95) |  | 0.040 |  |
| Regional LN recurrence |  | 34 | 63.0 |  | 20 | 37.0 |  | 1 |  | 0.015 |  |

iLRR isolated locoregional recurrence, SDR synchronous distant recurrence, NA not available, IDC invasive ductal carcinoma, ILC invasive lobular carcinoma, DCIS ductal carcinoma in situ, ER estrogen receptor, PR progesterone receptor, HER2 human epidermal growth factor receptor 2, TN triple negative6, RFI recurrence free interval, IBTR in breast tumor recurrence, LN lymph node, OR odds ratio, CI confidence interval
